# Role of axillary sentinel lymph node biopsy in patients with pure ductal carcinoma in situ of the breast

**DOI:** 10.1186/1471-2407-5-28

**Published:** 2005-03-11

**Authors:** Giorgio Zavagno, Paolo Carcoforo, Renato Marconato, Zeno Franchini, Giuliano Scalco, Paolo Burelli, Paolo Pietrarota, Mario Lise, Roberto Mencarelli, Giovanni Capitanio, Andrea Ballarin, Maria Elena Pierobon, Giorgia Marconato, Donato Nitti

**Affiliations:** 1 Clinica Chirurgica II, University of Padova, Via Giustiniani 2, 35128 Padova, Italy; 2 Chirurgia Generale, University of Ferrara, Corso Giovecca 203, 44100 Ferrara, Italy; 3 Chirurgia Generale, Hospital of Venezia, Castello 6776, 30122 Venezia, Italy; 4 Chirurgia Generale I, Hospital "Borgo Trento", Piazzale Stefani 1, 37126 Verona, Italy; 5 Chirurgia Generale II, Hospital of Vicenza, Via Rodolfi 6, 36100 Vicenza, Italy; 6 Chirurgia Generale, Hospital of Conegliano, Via Bisagno 4, 31015 Conegliano, Italy; 7 Chirurgia Generale II, Hospital "Borgo Trento", Piazzale Stefani 1, 37126 Verona, Italy; 8 Anatomia Patologica, University of Padova, Via Gabelli 61, 35128 Padova, Italy; 9 Anatomia Patologica, Hospital of Venezia, Castello 6776, 30122 Venezia, Italy

## Abstract

**Background:**

Sentinel lymph node (SLN) biopsy is an effective tool for axillary staging in patients with invasive breast cancer. This procedure has been recently proposed as part of the treatment for patients with ductal carcinoma in situ (DCIS), because cases of undetected invasive foci and nodal metastases occasionally occur. However, the indications for SLN biopsy in DCIS patients are controversial.

The aim of the present study was therefore to assess the incidence of SLN metastases in a series of patients with a diagnosis of pure DCIS.

**Methods:**

A retrospective evaluation was made of a series of 102 patients who underwent SLN biopsy, and had a final histologic diagnosis of pure DCIS. Patients with microinvasion were excluded from the analysis. The patients were operated on in five Institutions between 1999 and 2004.

Subdermal or subareolar injection of 30–50 MBq of 99 m-Tc colloidal albumin was used for SLN identification. All sentinel nodes were evaluated with serial sectioning, haematoxylin and eosin staining, and immunohistochemical analysis for cytocheratin.

**Results:**

Only one patient (0.98%) was SLN positive. The primary tumour was a small micropapillary intermediate-grade DCIS and the SLN harboured a micrometastasis. At pathologic revision of the specimen, no detectable focus of microinvasion was found.

**Conclusion:**

Our findings indicate that SLN metastases in pure DCIS are a very rare occurrence. SLN biopsy should not therefore be routinely performed in patients who undergo resection for DCIS. SLN mapping can be performed, as a second operation, in cases in which an invasive component is identified in the specimen. Only DCIS patients who require a mastectomy should have SLN biopsy performed at the time of breast operation, since in these cases subsequent node mapping is not feasible.

## Background

Ductal carcinoma in situ (DCIS) is defined as the proliferation of malignant epithelial cells in the mammary ductal system, with no evidence of invasion of the basement membrane. By definition, the disease is localized to the breast, with no spread to regional nodes or distant sites.

In the pre-mammography era, DCIS was rarely diagnosed, accounting for only 1 to 2% of all breast cancers. The increasing use of screening mammography in recent years has resulted in a dramatic increase in the diagnosis of DCIS, which now accounts for roughly 20% of all mammographically detected cancers [1].

Axillary treatment in patients with a diagnosis of pure DCIS is controversial. By definition, pure DCIS is not invasive and cannot spread to the regional nodes, so axillary staging should not be indicated in DCIS patients. In practice, however, nodal metastases are found in 1 to 2% of patients with a diagnosis of pure DCIS, and this paradoxical condition usually depends on a focus of invasion being missed by the pathologist because of a sampling bias [2]. Before the introduction of sentinel lymph node (SLN) biopsy, it was widely agreed that axillary lymph node dissection (ALND) should be avoided in patients with DCIS, because of the low yield of positive findings in these cases and the significant morbidity associated with the procedure [3]. The introduction of SLN biopsy as a minimally invasive tool for staging the axilla led to a renewed interest in axillary staging for these patients, for two reasons. First, this procedure is far less invasive than ALND and has minimal morbidity and complications, so the cost/benefit ratio may be positive even if the likelihood of finding metastatic nodes is low. Second, SLN biopsy allows more accurate axillary staging than ALND specimen examination, because SLN can be thoroughly evaluated by serial sectioning and immunohistochemical methods. This has led to the increased detection of nodal micrometastases in patients with invasive breast cancer, and has raised the expectation that the rate of positive nodal findings in patients with DCIS could also be increased. Currently, the indications for SLN biopsy in patients with a diagnosis of pure DCIS are controversial.

The aim of the present study was to determine the prevalence of SLN metastasis in a multi-institutional series of patients with a diagnosis of pure DCIS in order to determine the usefulness of routine SLN biopsy in such cases.

## Methods

### Patients

Our series consisted of 102 patients with a final histopathological diagnosis of pure DCIS who underwent SLN biopsy in five Institutions between January 1999 and January 2004. Patients with DCIS with microinvasion were excluded from the study.

All patients underwent SLN biopsy after they had given their written informed consent, and had decided whether they would undergo a completion ALND if SLN were found to be positive.

### Lymphoscintigraphy

On the day before surgery, all the patients received an injection of 30–50 MBq of 99 m-Tc-nanocolloidal albumin (Nanocoll, Nicomed-Italia, Saluggia, Italy) in 0.2 cc of saline.

In patients with palpable tumours, the radiocolloid was injected subdermally into the cutaneous projection of the tumour. In those with non-palpable tumours, the lesion was localized by stereotactic or ultrasonographic placement of a self-retaining wire and the cutaneous projection was marked by an ink spot on the skin, which served as a guide for subdermal radiocolloid injection. Patients with extensive microcalcifications or multifocal tumours were given subareolar radiotracer injection. Finally, patients who underwent delayed SLN biopsy after excision of the primary lesion were given subdermal radiotracer injection in two fractions, at the sides of the surgical scar.

Twenty minutes and 2 hours after injection, scintigraphic images were obtained using a large-field-of-view gamma camera (Orbiter, Siemens, IL, USA) equipped with a parallel hole, low energy and a high resolution collimator.

### Surgical procedure

Primary tumour resection with simultaneous SLN biopsy was performed in patients with a preoperative diagnosis of DCIS made by stereotactically-guided directional vacuum-assisted biopsy and in those with mammographical finding pathognomonic of DCIS, with or without a preoperative cytologic diagnosis of malignancy. A diagnostic excisional biopsy was performed in patients with a doubtful pre-operative diagnosis, and SLN biopsy was performed as a second operation in those with a final histologic diagnosis of DCIS.

As a rule, in patients undergoing conservative surgery for non-palpable lesions, an intraoperative x-ray examination of the resected specimen was performed, in order to confirm that complete tumour excision had been achieved.

SLN biopsy was performed 16 to 24 hours after radiocolloid injection. When conservative surgery was performed, the SLN was excised through the incision used for resecting the primary tumour, if located in the upper outer quadrant, whereas a separate axillary incision was performed if the tumour was located in another breast site. A gamma-ray-detecting probe was used for intraoperative SLN identification. All lymph nodes with a probe-detected radioactive count >10% of that of the hottest node were excised.

### Histopathology

The excised breast lesions were sampled with serial cuts and the margins were identified by ink.

The histopathologic diagnosis and classification of DCIS was made according to the Holland grading system [4]. The Van Nuys prognostic index [5] was used only in some of the cases. The search for microinvasive foci was performed in selected cases both with haematoxylin-eosin serial sections and immunostains to smooth muscle actin and CD10 for the detection of myoepithelial cells. In all cases, the tumour size and margin status were specified in the histopathological report. The SLN examination was performed as described elsewhere [6]. Briefly, for frozen-section examination, nodes with diameters of ≤ 0.5 cm were bisected, while nodes measuring >0.5 cm were sectioned each 2 to 3 mm. For each sample, two frozen sections made at 40 μm intervals were examined. The frozen tissue was then thawed, fixed and embedded to obtain permanent sections.

For definitive histological examination, two consecutive 5 μm thick tissue sections were cut from a paraffin block at two levels, 40 μm apart from each other. The sections were then stained with haematoxylin-eosin and immunostained with monoclonal antibody to cytokeratin.

## Results

The 102 patients with pure DCIS had a median age of 59.4 years (range 37 to 85). The histologic characteristics of the tumours are reported in Table 1.

Of the 102 patients, 20 (19.6%) had palpable, and 82 (80.4%) non-palpable breast tumours.

Seventy-four patients (72.5%) were treated with conservative surgery (quadrantectomy or lumpectomy) and 28 (27.5%) with mastectomy.

Ninety-one patients underwent primary tumour resection with simultaneous SLN biopsy: 16 on the basis of a preoperative histologic diagnosis of DCIS obtained by a vacuum-assisted large core biopsy, 63 with a preoperative cytology suggestive of malignancy and 12 only on the basis of the mammographical findings alone.

Patients with a doubtful preoperative diagnosis underwent excisional biopsy of the breast lesion, and the 11 patients with pure DCIS at definitive histology, underwent SLN biopsy and, if required, definitive treatment of the primary tumour as a second operation.

A total of 147 SLNs were identified and excised from the 102 patients: a single SLN was found in 61 cases (59.8%), two SLNs in 37 (36.3%), and three SLNs in 4 (3.9%).

A positive SLN was found in one (0.98%) patient, who had one micrometastases (0.6 mm) detected by haematoxylin-eosin staining. The pathologic review of the surgical specimen confirmed that the tumour had been completely excised, and no microinfiltration was detected. In this SLN positive case, the primary tumour was a micropapillary DCIS, G2, with a diameter of 16 mm. The patient did not undergone previous microbiopsy or FNAC, and SLN biopsy was performed at the time of the primary tumour excision. Completion ALND was not performed.

## Discussion

Indications for routine SLN biopsy in patients with a diagnosis of pure DCIS are still controversial. The first study proposing SLN biopsy for DCIS patients came from the H. Lee Moffitt Cancer Center in the year 2000 [7]: in a study of 87 DCIS patients, 5 (5.7%) presented SLN metastases, and the authors concluded that SLN biopsy should be routinely used in DCIS patients in order to identify and correctly stage patients with undetected invasive disease. In a later report on 195 DCIS patients from the same Institution, 26 (13%) patients were SLN positive and this finding strengthened the recommendation that SLN biopsy should become a routine part of surgical treatment for all DCIS patients [8].

The Memorial Sloan-Kettering Cancer Center group reviewed patients with "high risk" DCIS and found that 9/76 (12%) patients had SLNs positive for metastases. The authors suggested that SLN biopsy should be performed in all DCIS patients with one or more of the following characteristics: palpable or mammographic mass, suspicion of microinvasion, multicentric disease, high nuclear grade or necrosis [9].

On the other hand, in subsequent studies negligible rates of nodal involvement were found in patients with pure DCIS: Kelly et al. [10] found nodal metastases in only 3/134 (2.2%) patients, Intra et al. reported SLN metastases in 7/223 (3.1%) patients [11], Farkas et al. found no cases of SLN metastasis among 44 patients [12]. All these investigators concluded that SLN biopsy should not be routinely performed in patients with DCIS, with the exception of those undergoing mastectomy, because this operation precludes the possibility of performing a subsequent SLN biopsy if invasive foci are found at histology of the mastectomy specimen. Our results are consistent with the latter series of reports, since we found only one case of SLN micrometastasis among 102 patients with a diagnosis of pure DCIS (0.98%).

The variability in the reported rates of nodal metastases probably reflects differences in the accuracy of pathological evaluation of the primary breast tumour: extensive sampling and a thorough histological examination of the DCIS are of crucial importance in ruling out microinvasive foci. Microinvasive DCIS is a different pathological entity, with a well-defined metastatic potential, and should be excluded from studies evaluating the role of axillary staging in non-invasive cancer.

Our results support the view that the presence of axillary nodal metastases in patients with a final histopathological diagnosis of pure DCIS is a very unusual phenomenon, if the primary breast tumour has been completely excised and thoroughly examined by the pathologist. We, therefore, believe that routine SLN biopsy in all DCIS patients represents an over-treatment and should be avoided.

Enthusiasm for SLN biopsy in DCIS patients is partly due to the low morbidity of the procedure. However, complications such as lymphedema, seroma, infection and sensory neuropathy have been reported after SLN biopsy [13-15]. Moreover, performing SLN biopsy in patients with a small DCIS treated with a conservative operation precludes the possibility of using this procedure in patients with a subsequent homolateral invasive tumour, which is not infrequent in this setting [1].

Finally, the policy of performing primary tumour excision with simultaneous SLN biopsy in all patients with a preoperative diagnosis of DCIS may incur a risk of performing axillary biopsy in patients with benign breast lesions, since intraoperative frozen section histology is usually unreliable in patients with small areas of microcalcifications.

We therefore believe that this procedure should be used cautiously, being reserved for cases in which a real advantage can be expected.

Another factor that has prompted interest in SLN biopsy for DCIS patients is the widespread use of image-guided core needle biopsy for the diagnosis of mammographically-detected abnormalities, a diagnostic technique that often fails to identify invasive foci: 14 to 29% of patients with a preoperative core needle biopsy of DCIS are found to have invasive cancer at surgical excision [16,17], and require a second operation for axillary staging. With vacuum-assisted large core biopsy the under-diagnosis rate is lower, but it is still not negligible. Supporters of routine SLN biopsy claim that this procedure is not reliable after primary tumour excision. They therefore believe that all patients with a preoperative diagnosis of DCIS who undergo definitive surgery and are upstaged because the pathologist finds foci of invasion on the specimen, must undergo an ALND if a SLN biopsy was not performed at the time of the first operation [18]. We disagree with this view, because it has been clearly demonstrated that SLN biopsy can be safely performed as a second procedure after primary tumour excision [19,20]. The only exceptions to this are patients who undergo mastectomy and those who require a wide quadrantectomy of the upper outer quadrant, which can disrupt lymphatic pathways toward the axilla. We therefore believe that only patients with a preoperative diagnosis of DCIS who need mastectomy or wide excision close to the axilla should undergo a concomitant SLN biopsy. In all other cases, only the primary tumour excision should be performed and SLN biopsy should be reserved, as a second procedure, for patients found to have infiltration at the histologic examination of the primary tumour.

It has been claimed that some features of DCIS (large dimensions, high grade, comedo-type, mass forming lesions) are associated with a higher risk of invasive disease and nodal metastases. Therefore, some authors suggest that SLN biopsy should be reserved for all patients with these "high- risk" DCIS [9,21,22]. However, several investigators fail to correlate any histopathologic parameter with lymph node metastases [8,10,11].

The majority of patients in our series presented with low risk DCIS: most of the tumours were small (<1 cm) and only 20/102 patients had a palpable breast mass. Therefore, we cannot rule out that a higher incidence of microinvasion and nodal metastases might be found in a patient population with more aggressive forms of DCIS. However, in our series, the one DCIS patient with SLN metastasis had a small micropapillary, G2, non-palpable tumour.

In our opinion, clinical judgement should be used in patients with large solid tumours or diffuse comedo-type DCIS, bearing in mind that most of these patients require a mastectomy and are therefore candidates for SLN biopsy.

## Conclusion

Our results confirm that the finding of SLN metastasis in pure breast DCIS is a very rare occurence, if the primary tumour has been completely excised and microinvasion has been ruled out by a thorough histologic examination. Therefore, our current policy is to avoid routine SLN biopsy at the time of primary tumour resection in the presence of a preoperative diagnosis of DCIS. We reserve this procedure, as a second operation, for cases in which an invasive component is identified at the histologic examination of the surgical specimen. Only patients with a diagnosis of DCIS requiring a mastectomy or a wide resection close to the axilla should undergo concomitant SLN biopsy.

However, particularly if a large high-grade tumour is found, patients should be informed of the risk that invasive disease may be found, and that a second procedure for SLN biopsy may be required. In these cases, the patient can decide whether to undergo a potentially unnecessary SLN biopsy at the time of the first operation or whether to run the risk of requiring a second operation.

## Competing interests

The author(s) declare that they have no competing interests

## Authors' contributions

GZ planned the study and drafted the manuscript

PC, RM, ZF, GS, PB, PP, AB contributed with their own cases to the final multi-institutional series

RM and GC reviewed the pathologic specimens of selected cases and drafted the histopatology section of the manuscript

MEP and GM were in charge of data collection

ML and DN co-ordinated the study

## Pre-publication history

The pre-publication history for this paper can be accessed here:



## Figures and Tables

**Table 1:** Characteristics of pure DCIS diagnosed in 102 patients

	PATIENTS	
	N	%
HISTOLOGIC SUBTYPE		
Comedo	42	41.2
Cribriform	16	15.7
Solid	17	16.7
Papillary	7	6.9
Micropapillary	6	5.9
Comedo and solid	5	4.9
Others	9	8.8
		
TUMOUR SIZE		
0–5 mm	14	13.7
5–10 mm	40	39.2
10–15 mm	19	18.6
15–20 mm	14	13.7
20–30 mm	10	9.8
>30 mm	2	2.0
Unknown	3	2.9
		
TUMOUR GRADE		
G1	21	20.6
G2	37	36.3
G3	44	43.1
		
TYPE OF SURGERY		
Lumpectomy/quadrantectomy	74	72.5
Mastectomy	28	27.5
